# Self-Assembly Synthesis of Silver Nanowires/Graphene Nanocomposite and Its Effects on the Performance of Electrically Conductive Adhesive

**DOI:** 10.3390/ma11102028

**Published:** 2018-10-18

**Authors:** Tao Xu, Jiayu Chen, Wenhui Yuan, Yinhua Liu, Yongjun Sun, Huijun Wu, Xiaoqing Zhou

**Affiliations:** 1 Academy of Building Energy Efficiency, School of Civil Engineering, Guangzhou University, Guangzhou 510006, China; xutao9@mail.sysu.edu.cn (T.X.); wuhuijun@tsinghua.org.cn (H.W.); zhou_xiaoqing03@163.com (X.Z.); 2 Department of Architecture and Civil Engineering, City University of Hong Kong, Tat Chee Avenue, Kowloon, Hong Kong; jiaychen@cityu.edu.hk; 3 School of Chemistry and Chemical Engineering, South China University of Technology, Guangzhou 510640, China; jinyinhua0520@163.com; 4 Division of Building Science and Technology, City University of Hong Kong, Tat Chee Avenue, Kowloon, Hong Kong; yongjsun@cityu.edu.hk

**Keywords:** electrically conductive adhesive, silver nanowires, graphene, volume resistivity

## Abstract

Among recent advances in electronic packaging technologies, electrically conductive adhesives (ECAs) attract most researchers’ attention, as they are environment-friendly and simple to apply. ECAs also have a lower operating temperature and volume resistivity compared with conventional electronic conductive adhesives. In ECAs, the conducting fillers play a significant role in improving conductivity and strength. In this work, as filler additives, the silver nanowires/graphene nanocomposites (AgNWs-GNs) were successfully fabricated via a facile self-assembly method. The characteristics of the as-prepared nanocomposites were evaluated by FTIR (Fourier Transform infrared spectroscopy), XRD (X-ray Diffraction), XPS (X-ray photoelectron spectroscopy), TEM (Transmission electron microscope) and Raman tests, demonstrating a successful synthesis process. Different amounts of AgNWs-GNs were used as additives in micron flake silver filler, and the effects of AgNWs-GNs on the properties of ECAs were studied. The results suggested that the as-synthesized composites can significantly improve the electrical conductivity and shear strength of ECAs. With 0.8% AgNWs/GNs (AgNWs to GO (Graphite oxide) mass ratio is 4:1), the ECAs have the lowest volume resistivity of 9.31 × 10^−5^ Ω·cm (95.4% lower than the blank sample without fillers), while with 0.6% AgNWs/GNs (AgNWs to GO mass ratio is 6:1), the ECAs reach the highest shear strength of 14.3 MPa (68.2% higher than the blank sample).

## 1. Introduction

Traditionally, Sn-Pb solder is widely applied on electronic packaging for its high conductivity, low melting point, and low cost [1,2]. However, Sn-Pb solder is highly toxic to both human health and the environment. In addition, Sn-Pb soldering requires a high operating temperature but has low resolution. Compared to Sn-Pb solder, electrically conductive adhesives (ECAs) are regarded as the optimal alternative electronic conductive materials, since they are more environment-friendly and convenient during application with lower operating temperatures [3,4,5,6,7]. ECAs are produced by introducing electrically conductive fillers into an organic polymer matrix to form a uniformly dispersed mixture [8], which contains a rich resin matrix, curing agent, curing accelerator, diluent, and toughening agent [9]. ECAs have excellent mechanical and adhesive properties due to various resin matrices, such as epoxy resin, phenolic resin, acrylate, polyurethane, silicone resin and polyimide resin. As another critical content of ECAs, conductive fillers fall into three material categories, including metal [10,11], metallic oxide, and carbon material [12]. These fillers are dispersed in resin matrix as flaky, granular, dendritic, and rod-like shapes, forming the conductive pathway for ECAs’ strong conductivity. 

After the discovery of ECAs, researchers have invested a great deal of effort into improving ECAs’ conductivity through various strategies, such as increasing the shrinkage rate of epoxy resin, surface modification of flake silver powder, introducing glow-melting-point metal components and incorporating nanofillers. Nanofillers can improve the conductivity of ECAs by reducing contact resistance or tunneling resistance. Recently, researchers reported that electrical conductivity can be developed by adding nano ball-shaped silver powder and micro-sized silver powder [13,14,15]. Lee et al. demonstrated that silver nanoparticles can improve electrical conductivity when the volume fraction is lower than the percolation threshold [16]. Jiang et al. showed in their research that when the total mass fraction of silver flakes epoxy resin fillers with silver nanoparticles is 80%, ECAs have the lowest volume resistivity of 5.0 × 10^−5^ Ω·cm [13]. Other researchers tried to in-situ synthesize Ag nanoparticles in the curing process of epoxy resin to eliminate residual Ag nanoparticles during low-temperature sintering. Gao et al. found that cured ECAs filled with the in-situ synthesized Ag nanoparticles in epoxy resin have a high electricity conductivity with a volume resistivity as low as 7.5 × 10^−5^ Ω·cm [17]. To achieve this result, the curing process should exclude any curing agent except 2-ethyl-4methyl imidazole (EMZ) using the thermal decomposition of silver acetate-imidazole complex under the molar ratio of 2:1. 

According to these studies, it is difficult for ECAs to have a volume resistivity lower than 10^−4^ Ω·cm, but a lower value can be achieved through adding silver nanowires filler with proper aspect ratio (height/diameter) [18]. Compared with spherical silver nanowires, filler with a higher aspect ratio (height/diameter) at low contents can form a percolated network that enlarges the contact area for electron transportation, then the contact resistance can be reduced [19,20]. Wu et al. reported that ECAs with silver nanowire fillers have less mass fraction (56%) than traditional micron and silver nanoparticles and can reach lower volume resistivity (1.2 × 10^−4^ Ω·cm) and higher shear strength (17.6 MPa) [21]. Yu et al. also found that ECAs filled with 50% (mass fraction) silver nanowires, modified with APTS (3-Aminopropyltriethoxysilane) on the surface, have greatly improved conductivity [22]. Chen et al. studied the resistivity of the micron-silver-composed ECAs and silver nanowires blended ECAs and concluded that the conductivity of the former is better when the other contents are the same [23]. The impact of single silver nanowires and the complex packing of silver nanowires and nanoparticles on the electrical conductivity of ECAs was reported by Chen et al. [24]. Wang et al. utilized the Ag nanowires (with a diameter of about 200–570 nm and a length of about 20–100 μm) and concluded that the conductivity of epoxy resin ECAs was evidently improved [25]. In 2016, Ma et al. also concluded that the conductivity of ECAs is efficiently improved with silver nanowires‒nanoparticles composite prepared using the hydrothermal method [26]. Xiao et al. discovered that, due to the sintering effect of Ag nanowires, when the content of functionalized-silver-nanowires/flake-silver-powder complex is 75% (mass ratio is 2:3), the volume resistivity can reach 5.8 × 10^−6^ Ω·cm [27].

Other preferable fillers for EACs are nanographites, such as graphene and carbon nanotube. Because of the large specific surface area and high aspect ratio, carbon nanotubes and graphene can help ECAs reach the percolation threshold. Carbon nanotubes are tubular graphite material and can be classified as single, double and multiple walled carbon nanotubes. Graphene has a two-dimensional honeycomb structure that is made up of single-layer carbon atoms [28]. When graphite is added, the density of ECAs drops sharply and its mechanic strength enhances significantly [29]. However, when introduced into resin based ECAs, nanographite only has limited impact on their strength [30]. Marcq et al. [31] found that when multiple-walled carbon nanotubes are dispersed in acetone with dispersant, the carbon nanotubes and flake Ag powder can generate a synergic effect to improve ECAs’ conductivity. Luan et al. prepared a new type of hybrid filler, which was composed of one-dimensional Ag nanowires and two-dimensional reduced graphene, and reported its conductivity was surprisingly improved [32]. Based on these conclusions, researchers also tried to implement silver nanoparticles to modify the surface of graphite so that the average conductivity and contact resistance of fillers can be further reduced [33]. Wu et al. [34] used surface-modified (with Ag silver nanoparticles) carbon nanotubes in epoxy resin and found that through adding the fillers, the conductivity of ECAs can reach 2.21 × 10^−4^ Ω·cm. Baik et al. [33] utilized Ni and Ag nanoparticles with acidified single-walled carbon nanotubes during surface modification and the commercial silver ECA samples had a significant improvement in conductivity. Zhao et al. [35] reported that the volume resistivity of ECAs using graphene, which were surface-modified using Ag nanoparticles, had mass fraction doped 1% and reached a conductivity of 4.6 × 10^−5^ Ω·cm (close to Sn/Pb solder).

Graphene has excellent electrical conductivity, thermal conductivity, and mechanic properties, but its non-hydrophilic and non-lipophilic properties constrain its application in ECAs. To address such constraints, researchers suggest graphene and nanomaterial mixtures as an alternative. There are two major methods to prepare graphene/inorganic nanomaterial composite: in-situ synthesis and self-assembly synthesis. In-situ synthesis forms the nanocrystal on the surface of graphene, and grows nanocrystals into nanoparticles, nanowires and nanorods. Self-assembly synthesis forms the nanomaterial first and then functionalizes the material to bond with graphene using a covalent bond or a non-covalent bond to overcome the insolubility of the nanocomposites. Compared with the in-situ synthesis, self-assembly synthesis is able to control the distribution, size, and load of nanomaterial on the surface of graphene. Self-assembly synthesis of silver nanowires/graphene nanocomposites (AgNWs–GNs) for ECAs was seldom reported up to now. Only in 2013, Tien et al. reported self-assembly AgNWs–GNs for transparent conductive films (TCFs) using NaBH_4_ as the reductant. However, the use of NaBH_4_ can lead to high cost. Therefore, this study prepared self-assembly AgNWs-GNs using low-cost hydrazine hydrate as the reductant. The results suggested that composites significantly improve the electrical conductivity and shear strength of ECAs. 

## 2. Experiment Design 

### 2.1. Raw Materials and Experiment Equipment

The raw materials and equipment used in the experiment are listed in Table 1 and Table 2. 

### 2.2. Preparation of Composites

#### 2.2.1. Preparation of Silver Nanowires (AgNWs)

First, polyvinylpyrrolidone (PVP) is dissolved in a 25 mL ethanediol solution of FeCl_3_ (concentration of 0.1 mmol/L). An AgNO_3_ solution (concentration of 0.1 mol/L) is added into the ethanediol solution drop by drop with a 15 min magnetic stir. The mixed solution is then moved to a reactor within an oven at a temperature of 160 °C for 25 h. After the completion of the reaction, the reactor is removed and cooled to room temperature. Finally, the crude products are washed with acetone several times.

AgNWs are prepared after 24 h vacuum drying of the centrifuged products [36]. Then, 369 mg of AgNWs and 1.3155 g of β-cysteamine are added into 250 mL of absolute ethyl alcohol. After 20 h of high-speed mechanical agitation under room temperature, the products are centrifuged and dried in a vacuum. Finally, amino functionalized AgNWs can be obtained [37] and marked as NH_2_-AgNWs.

#### 2.2.2. The Preparation of AgNWs/GO Composites

First, 50 mg of graphite oxide (GO) is dispersed into 200 mL of deionized water with 1 h supersonic dispersion. Then, 100, 200, 300 and 400 mg of NH_2_-AgNWs (Nano silver wires with amino functionalization) is added into the solution as four samples. After the 24 h mechanical agitation at room temperature, the products are washed with absolute ethyl alcohol water several times, and then the products are centrifugalized and dried. Finally, the AgNWs/GO nanocomposites obtained from the four samples are marked as AgNWs/GO-1, AgNWs/GO-2, AgNWs/GO-3 and AgNWs/GO-4.

#### 2.2.3. The Preparation of AgNWs/GNs

First, 100 mg of AgNWs/GO composites is dispersed into 100 mL of deionized water with 1 h supersonic dispersion. Then, 0.1 g KOH and 1 mL hydrazine hydrate is added into the solution for a 24 h reaction under 100 °C. The crude products are washed with absolute ethyl alcohol and deionized water several times and dried in a vacuum for 24 h at 60 °C. The final samples, AgNWs/GNs, are marked as AgNWs/GNs-1, AgNWs/GNs-2, AgNWs/GNs-3, and AgNWs/GNs-4.

#### 2.2.4. Preparation of Conductive Adhesive

The ECAs were mainly composed of resin matrix, micron silver flake fillers and AgNWs-GNs additives. First, epoxy resin, methyl hexahydrophthalic anhydride and 2-ethyl-4-methylimidazole are mixed as the resin matrix according to the mass ratio of 1:0.85:0.0185. Then, micron silver flakes (MSF, 10–20 μm) are added into the resin matrix. The silver nanowires/graphene composites are used as additives in the MSF filler. The total mass fraction of the fillers is 70%, while the mass fractions of the silver nanowires/graphene composite samples are 0.0% (blank), 0.2%, 0.4%, 0.6%, 0.8%, and 1.0% in fillers, respectively. After stirring for 1 h, the filler is evenly dispersed in the matrix and then the conductive adhesive sample is obtained. The sample is coated on the glass plate and cured for 2 h at 150 °C in the oven. Finally, the samples are ready for the experiment after cooling. 

### 2.3. Experiment Tests

The performance of ECAs was evaluated by shear strength and shear electrical conductivity tests. Every test was repeated 5 times, and the average value from the 5 results was adopted. The errors were calculated by the difference value between the maximum (or minimum) and average values.

#### 2.3.1. Tests on Shear Strength

The conductive adhesive sample was glued between two LY12-CZ aluminum alloy sheets for three samples with thicknesses of 2 ± 0.1 mm, 25 ± 0.2 mm, and 100 ± 0.2 mm. The width of the bonding part of two aluminum alloy sheets in the sample was 25 ± 0.2 mm and its length was 12.5 ± 0.5 mm. After curing and cooling to room temperature, the samples were pulled at a speed of 5 mm/min in parallel with the electronic universal material testing machine. The shear strength value can be calculated with

(1) $$\tau =\frac{f}{a}$$

where τ is the tensile shear strength (MPa); f is the maximum load (N); a is the overlap area (mm^2^). 

#### 2.3.2. Tests on Shear Electrical Conductivity

To prepare the conductivity test, two parallel tapes were pasted on the slides with a size of 25.4 mm × 76.2 mm. The prepared conductive adhesive was filled into the gap between the two tapes, evenly coated with the scraper, and then the tapes were removed. After curing and cooling to room temperature, the volume resistivity ρ (Ω·cm) of the conductive adhesives was measured using an RTS-9 double electric logging four-point probe tester.

## 3. Results and Discussion

ECAs with Ag wire fillers are widely used in microassembly circuits, electronics, and printed circuits. Due to the high price, the amount of Ag should be kept at a lower content in ECAs. Furthermore, the content must reach the percolation threshold. Therefore, AgNWs/GNs with high performances are essential for ECAs.

### 3.1. FTIR (Fourier Transform Infrared Spectroscopy) Spectra Analysis

**Figure 1:**
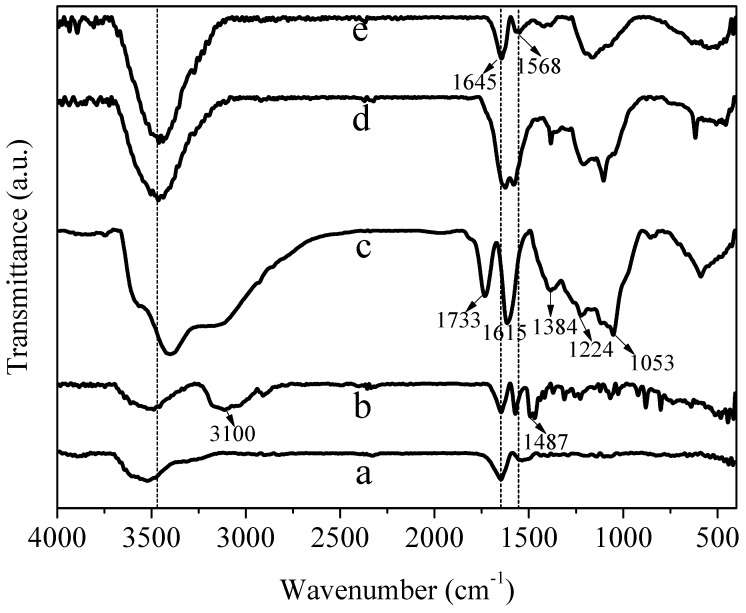
FTIR spectra of (**a**) silver nanowires (AgNWs); (**b**) NH_2_-AgNWs; (**c**) graphite oxide (GO); (**d**) AgNWs/GO-2 and (**e**) AgNWs/GNs-2.

Figure 1 shows the FTIR spectrum of AgNWs, NH_2_-AgNWs, GO, AgNWs/GO-2 and AgNWs/GNs-2. Compared with AgNWs, after modification, the NH2-AgNWs displays peaks at 3100 and 1487 cm^−1^, which could be due to the stretch and bend vibration of the N-H group, thus indicating that mercaptoethylamine had been successfully grafted onto the surface of silver nanowires. In Figure 1c, after oxidation of graphite, there was a -OH stretching vibration peak, a C=O stretching vibration peak of the carboxyl group (-COOH), an O-H vibration peak of the hydroxyl group, a C-O-C stretching vibration peak of the epoxy group, and a C-O vibration peak of the epoxy group at 3403, 1733, 1615, 1384 and 1053 cm^−1^. After graphite oxide was compounded with NH_2_-AgNWs, the stretching vibration peak of -NH_2_ disappeared, suggesting that graphite oxide and AgNWs formed AgNWs/GO composites through -C-N bonding. The NH_2_–AgNWs have many -NH_2_ functional groups, which can react with the epoxy (-C-O), hydroxyl (-OH) and carboxyl (-COOH) groups in GO to yield –C-N bonding. This process indicates that the AgNWs can covalently bond with GO [37,38]. After reduction, the absorption peaks of graphite oxide at 1733, 1615, 1384 and 1053 cm^−1^ were obviously weakened or even disappeared. Specifically, AgNWs/GNs-2′s absorption peak at 3403 cm^−1^ (-OH) is stronger than AgNWs/GO-2’s, which is different from the results of Sookhakian et al. This may be due to the interaction between -NH2 and -OH, leading to a stable -OH under reduction [39].

### 3.2. X-ray Diffraction Analysis

**Figure 2:**
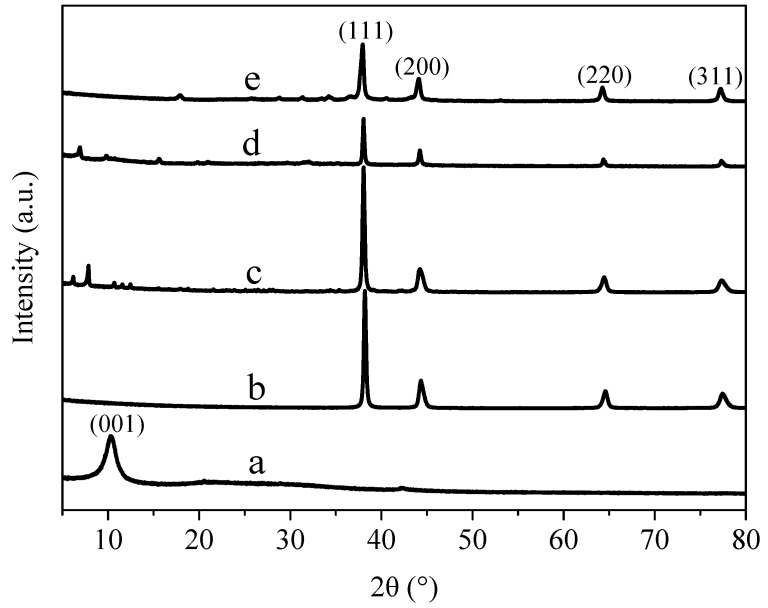
XRD patterns of the samples: (**a**) GO; (**b**) AgNWs; (**c**) NH_2_-AgNWs; (**d**) AgNWs/GO-1 and (**e**) AgNWs/GNs-1.

Figure 2 shows the XRD diagram of GO, AgNWs, NH_2_-AgNWs, AgNWs/GO-1 and AgNWs/GNs-1. As shown in Figure 2 curve a, a diffraction peak (001) appeared at 10.28°, suggesting GO was successfully prepared. In Figure 2 curve b, there were four diffraction peaks appearing at 2θ values of 38.2°, 44.4°, 64.5°, and 77.5°, which correspond to (111), (200), (220), and (311) crystal planes of the silver face-centered cubic system, respectively. These peaks are consistent with the standard card of silver (JCPDS No.04-0783). When the silver nanowires were modified with mercaptoethylamine, the diffraction peaks of NH_2_-AgNWs shifted to a lower angle than those of pure silver nanowires. This means that the mercaptoethylamine molecule was hydrolyzed and a small amount of S formed coordination bonds with Ag. When NH_2_-AgNWs were compounded with graphite oxide, a weak diffraction peak (001) appeared at 10.28°. After hydrazine hydrate reduction, the diffraction peak of graphite oxide disappeared, indicating that graphite oxide had been successfully reduced.

**Figure 3:**
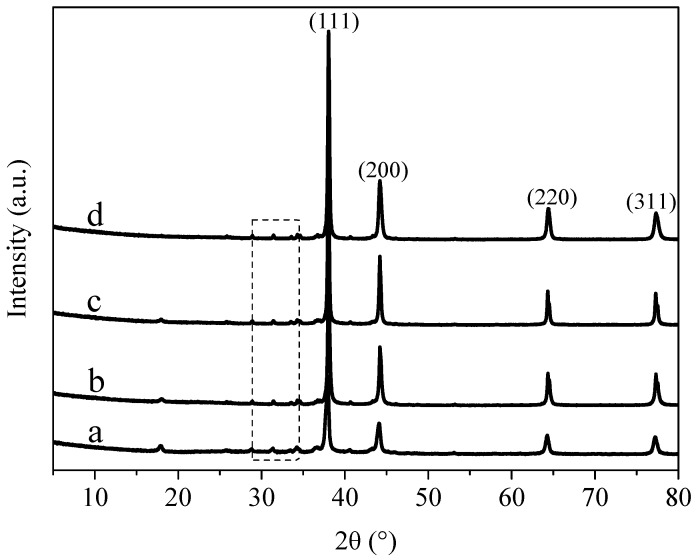
XRD patterns of the samples: (**a**) AgNWs/GNs-1; (**b**) AgNWs/GNs-2; (**c**) AgNWs/GNs-3 and (**d**) AgNWs/GNs-4.

Figure 3 shows the XRD pattern of AgNWs/GNs complex with different mass ratios of silver nanowires and graphite oxide. The figure shows that with the increase of the mass ratio of silver nanowires to graphite oxide, the positions of diffraction peaks corresponding to silver nanoparticles remains the same. This indicates that the interfacial spacing of silver nanoparticles is independent to the amount of silver particles added. A small intensity peak appeared at 31.4° and it belongs to the diffraction peak of silver oxide. With the increase of silver nanowires content, the diffraction peaks of the oxides almost disappeared, indicating that adding silver nanowires can improve the stability of AgNWs/GNs composite.

### 3.3. SEM and TEM Analysis

**Figure 4:**
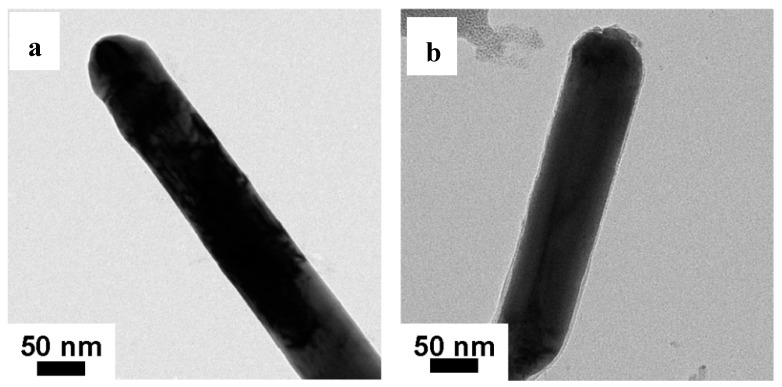

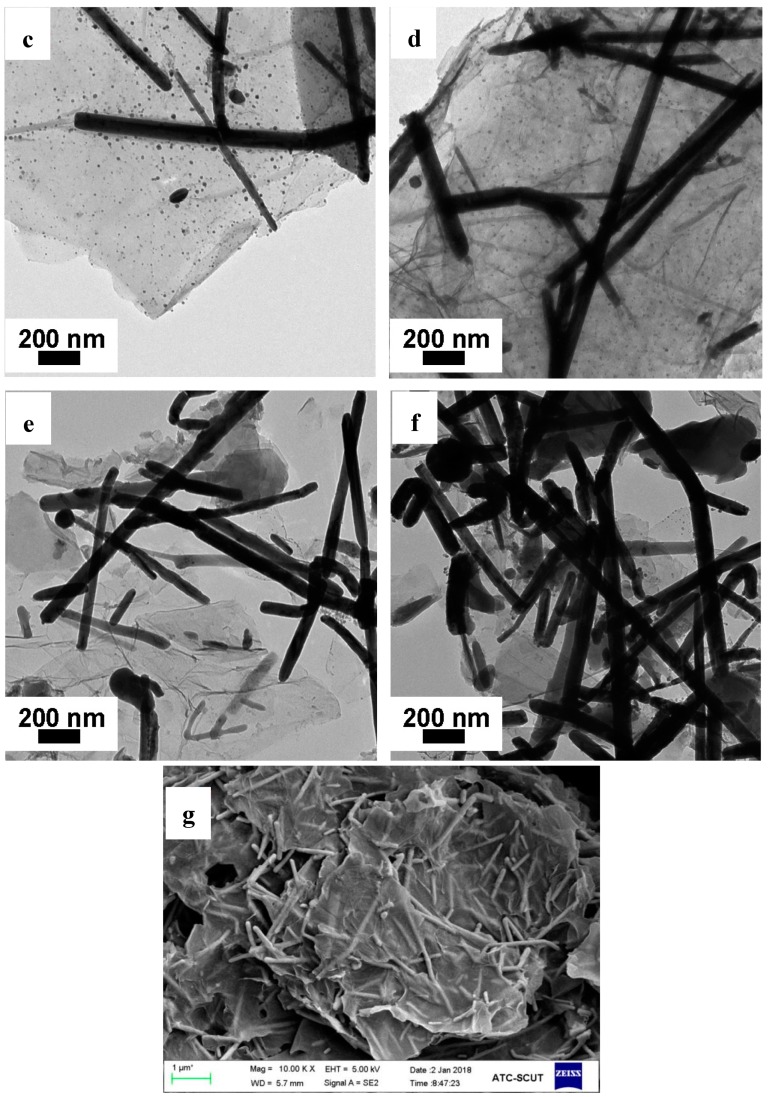
TEM images of the samples: (**a**) AgNWs; (**b**) NH2-AgNWs; (**c**) AgNWs/GNs-1; (**d**) AgNWs/GNs-2; (**e**) AgNWs/GNs-3 and (**f**) AgNWs/GNs-4; SEM images of (**g**) AgNWs/GNs-2.

Figure 4 shows the TEM diagram of AgNWs, NH_2_-AgNWs and AgNWs/GNs and SEM diagram of AgNWs/GNs-2. As shown in Figure 4a, the diameter of the silver nanowires is about 80 nm. After the silver nanowires were modified with mercaptoethylamine, mercaptoethylamine was grafted onto the surface of the silver nanowires using an S-H bond, resulting in NH_2_ functional groups attached to the surface of silver nanowires. As shown in Figure 4b, the surface of the silver nanowires was coated with a layer of mercaptoethylamine. The functionalized silver nanowires were connected to epoxy groups on the surface of graphite oxide by NH_2_. Figure 4c–f show the composite of silver nanowires and graphene. When the content of silver nanowires is low, the wires were sparsely distributed on the graphene lamellae. When the mass ratio of silver nanowires to graphite oxide is higher than 4:1, the distribution of silver nanowires embedded in the graphene surface becomes uniform. With the further increase of silver nanowires content, the silver nanowires will load on graphene lamellae agglomerates. The number of graphene layers in AgNWs/GNs composites was smaller because the silver nanowires prevent the agglomeration of graphene lamellae. It can be seen from Figure 4g that the silver nanowires are evenly distributed between the graphene sheets. 

### 3.4. Raman Spectra Analysis

**Figure 5:**
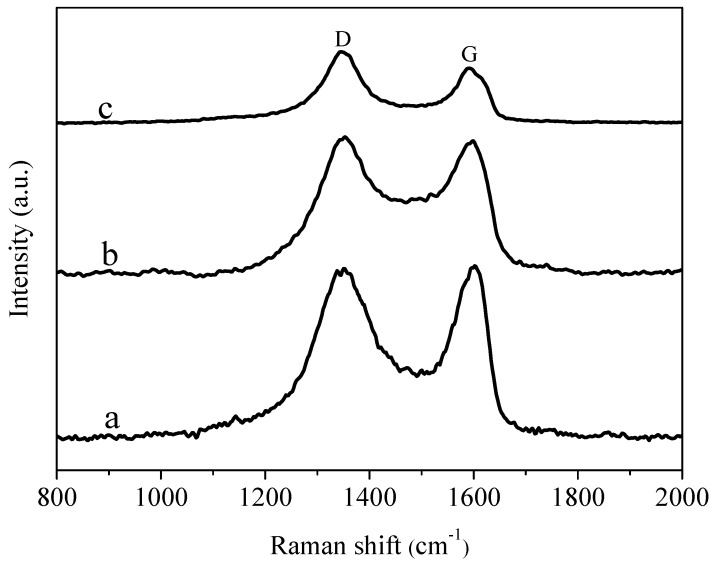
Raman spectra of the samples: (**a**) GO; (**b**) AgNWs/GO-2 and (**c**) AgNWs/GNs-2.

Figure 5 shows the Raman spectra of GO, AgNWs/GO-2 and AgNWs/GNs-2. The Raman spectrum of graphite oxide has two main peaks, D peak at 1350 cm^−1^ and G peak at 1600 cm^−1^, which represent the A1g phonon pattern and the E2g symmetric phonon vibration of the k point, respectively. The G band originates from the carbon vibration in the sp2 plane, and the D band is derived from the presence of structural defects, disorders and heteroatomic decoration in the graphene sheet. The integrated intensity ratio ID/IG is broadly applied for determining the defect quantity in graphitic materials [40]. The peak intensities ratio of the D peak to the G peak (ID/IG) is 0.98. When combining graphite oxide with the silver nanowires, the ID/IG of the AgNWs/GO composite is 1.01, which is higher than the ID/IG of the graphite oxide. This indicates an increased disorder in the graphene after the modification. The ID/IG ratio of AgNWs/GNs composite is 1.05, higher than that of graphite oxide and AgNWs/GO composite, suggesting a decrease in the average size and an increase in the number of sp^2^ domains upon reduction [41].

### 3.5. XPS (X-ray Photoelectron Spectroscopy) Spectra Analysis

**Figure 6:**
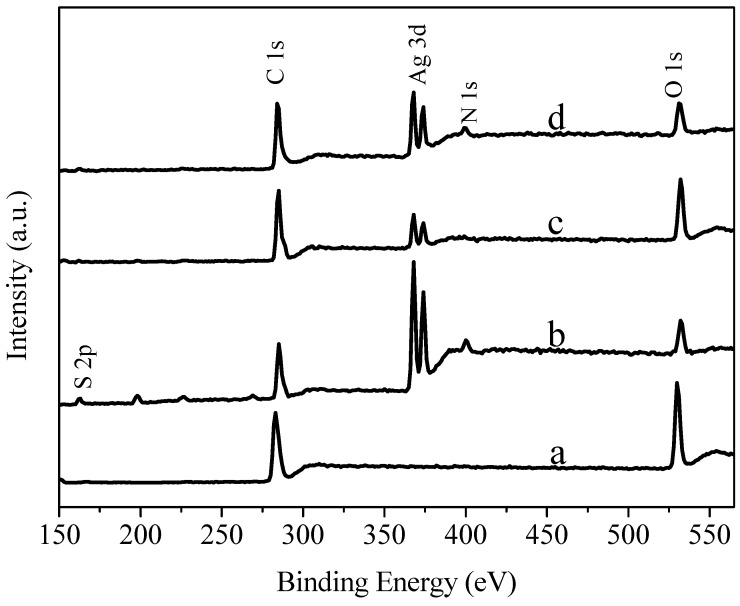
XPS (X-ray photoelectron spectroscopy) wide scans of (**a**) GO, (**b**) NH2-AgNWs, (**c**) AgNWs/GO-2 and (d) AgNWs/GNs-2.

Figure 6 shows the XPS wide scans of GO, NH_2_-AgNWs, AgNWs/GO-2, and AgNWs/GNs-2. As shown in the figure, GO mainly had carbon and oxygen elements, while NH_2_-AgNWs mainly had sulfur, carbon, silver, nitrogen and oxygen elements. This suggests that mercaptoethylamine had been successfully grafted onto the surface of the silver wires. The N1s signal is weakened and even disappeared after the silver wires were compounded with graphite oxide. This result suggests that the silver wires and graphite oxide were connected by NH_2_. The content of the oxygen element decreased after reduction of AgNWs/GO complex, meaning that graphite oxide had been successfully reduced.

**Figure 7:**
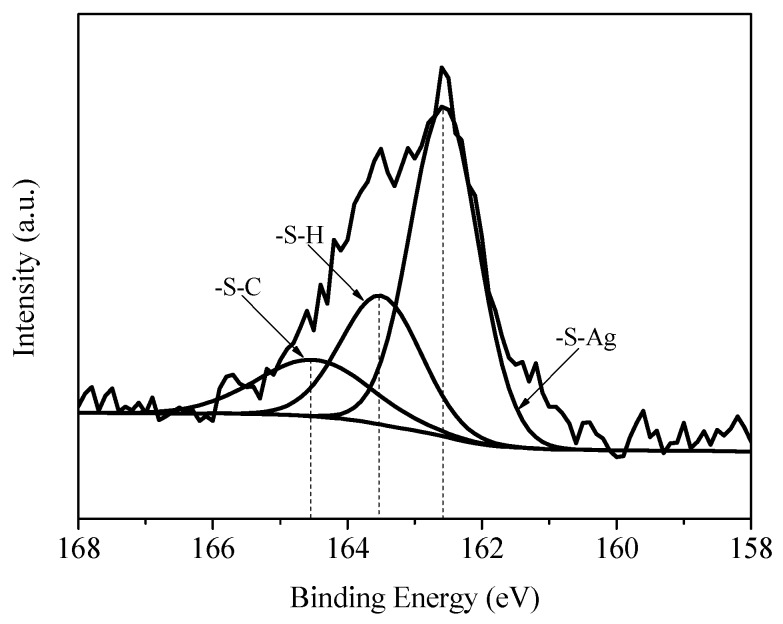
S 2p XPS spectra of NH2-AgNWs.

Figure 7 is the S 2p XPS spectrum of NH2-AgNWs. From the figure, it can be identified that the groups attached to the S atom mainly include -SC, -SH, and -S-Ag with corresponding binding energies of 164.5, 163.5 and 162.6 eV [37,38]. The -SH group was not bonded to the surface of the silver nanowires curve, but -S-Ag was bonded. According to the results, mercaptoethylamine was successfully grafted onto the surface of the silver nanowires.

**Figure 8:**
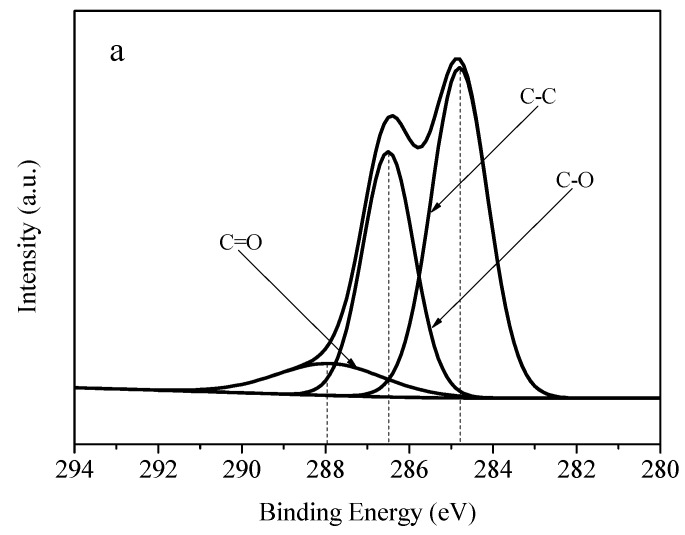

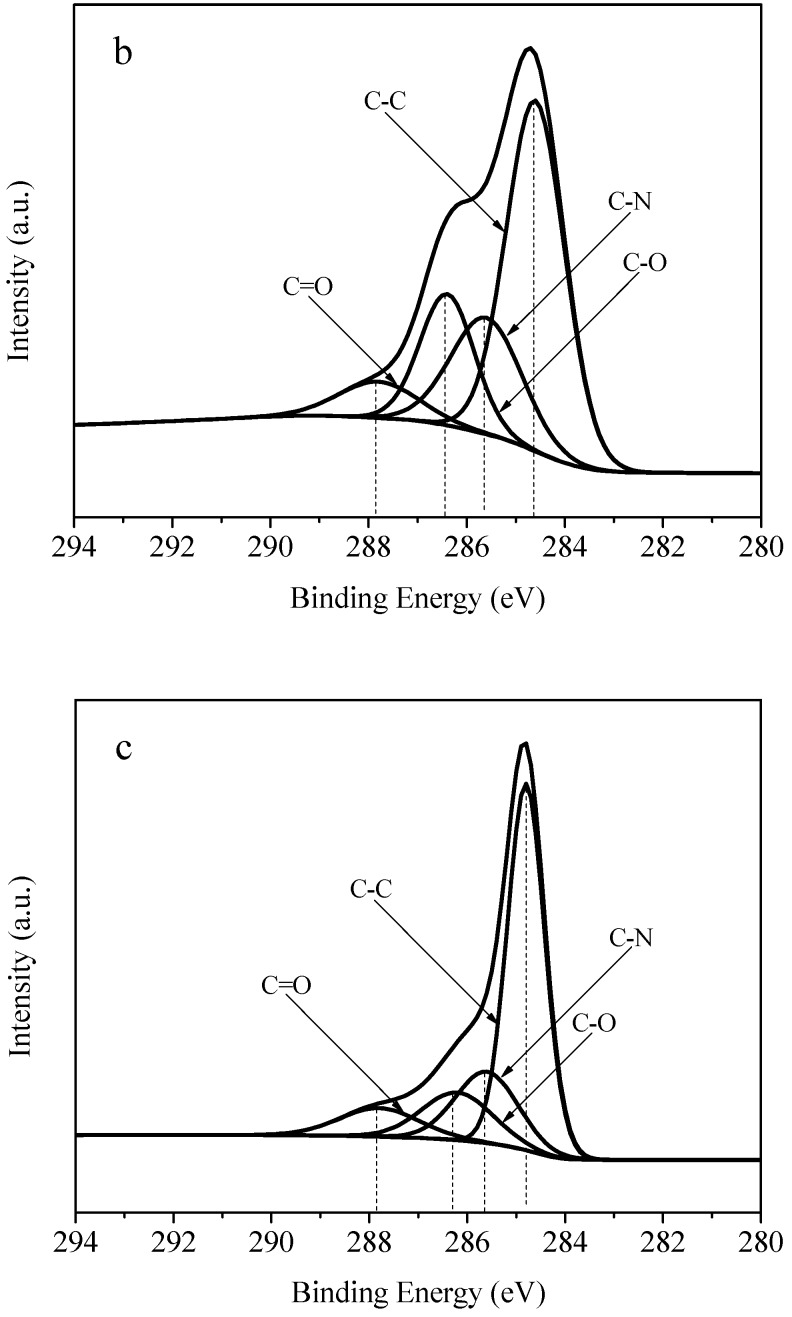
C 1s XPS spectra of (**a**) GO; (**b**) AgNWs/GO-2 and (**c**) AgNWs/GNs-2.

Figure 8 shows the C 1s XPS spectra of GO, AgNWs/GO-2, and AgNWs/GNs-2. Figure 8a shows that the C atom of the graphite oxide is mainly composed of C-C, C-O and C=O. The content of the oxygen-containing bond is high. Figure 8b suggests that, after the functionalized silver nanowires and graphite oxide were combined, the CO bond was reduced, and the CN bond was increased. It suggests the surface of the functionalized silver nanowires was attached with NH_2_ functional groups, which can react with the graphite oxide epoxy group to form a bond. This result supports the conclusions that the functionalized silver nanowires can be combined with the graphite oxide by covalent bond. When AgNWs/GO-2 is reduced, the C-O and C=O bonds can be greatly destroyed, and graphite oxide can be reduced as well. 

### 3.6. Performance Analysis

**Figure 9:**
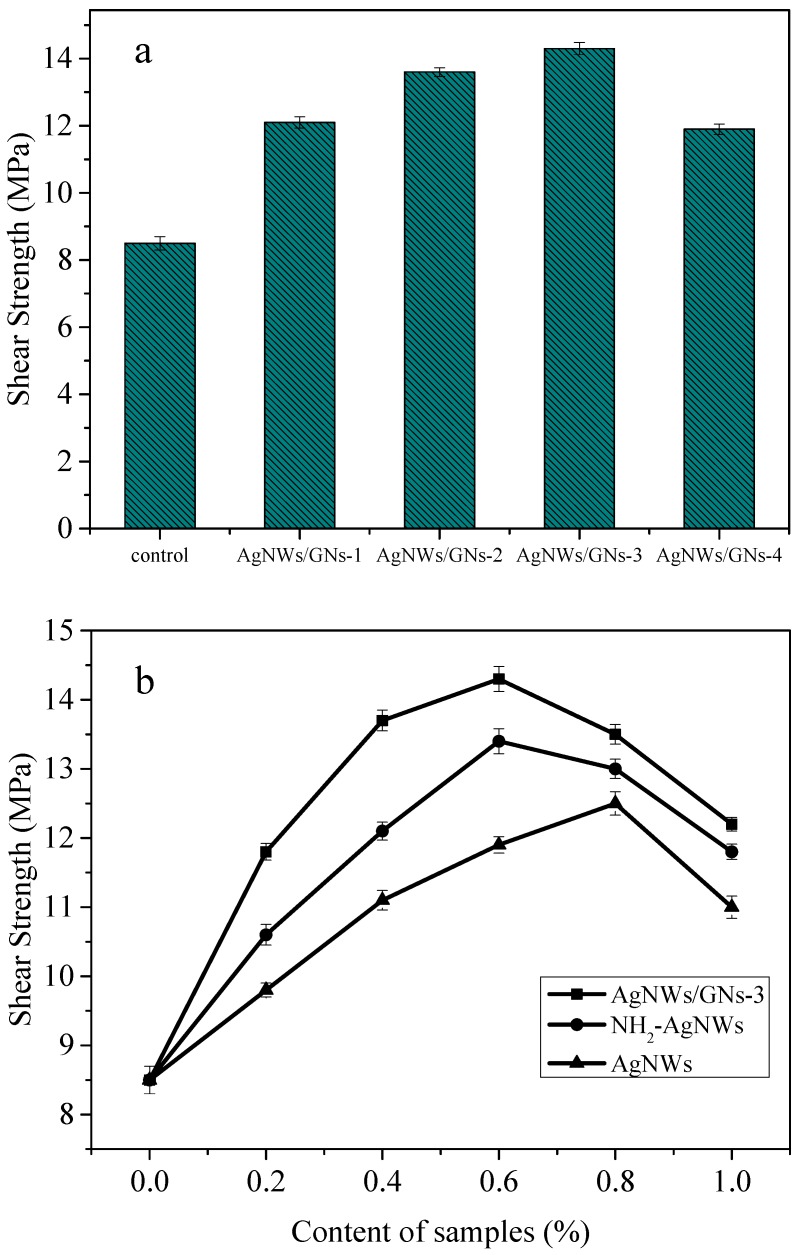
Shear strength of the ECAs filled with the nanocomposites with different proportions of AgNWs to GO (**a**) and different contents of the samples (**b**).

Figure 9a compares the shear strength of conductive adhesive with different silver nanowires to graphite oxide mass ratio. Figure 9b compares the shear strength of conductive adhesive with silver nanowires, functionalized silver nanowires, and AgNWs/GNs-3 as composite content. The blank conductive adhesive has a shear strength of 8.5 MPa. When the mass ratio of silver nanowires increases, the shear strength of the conductive paste increases first and then decreases. When the mass ratio of silver nanowires to graphite oxide is 6:1 (AgNWs/GNs-3), the shear strength of the conductive paste reaches its maximum at 14.3 MPa, 68.2% higher than the blank conductive adhesive. Figure 9b shows that adding AgNWs, NH_2_-AgNWs, and AgNWs/GNs-3 nano-fillers can effectively improve the mechanical properties of the conductive adhesive. When NH_2_-AgNWs and AgNWs with mass fractions of 0.6% and 0.8% are added, the shear strengths of the conductive paste reach 13.4 MPa and 12.5 MPa, respectively. This suggests that the dispersibility of the functionalized silver nanowires in the resin matrix is improved, and the interaction by the amine groups in AgNWs/GNs and the oxirane rings in the epoxy enhanced the interfacial adhesion between the silver nanowires and the resin matrix. The modified compatibility between the silver nanowires and the resin was also improved [42,43]. When AgNWs/GNs-3 with a mass fraction of 0.6% was added, the shear strength of the conductive paste reached its maximum at 14.3 MPa. After compounded with functionalized silver nanowires, graphene would synergistically enhance the shear strength of the conductive adhesive. However, when there is too much nanocomposite, agglomeration would occur in the epoxy resin matrix and cause the shear strength to decrease.

**Figure 10:**
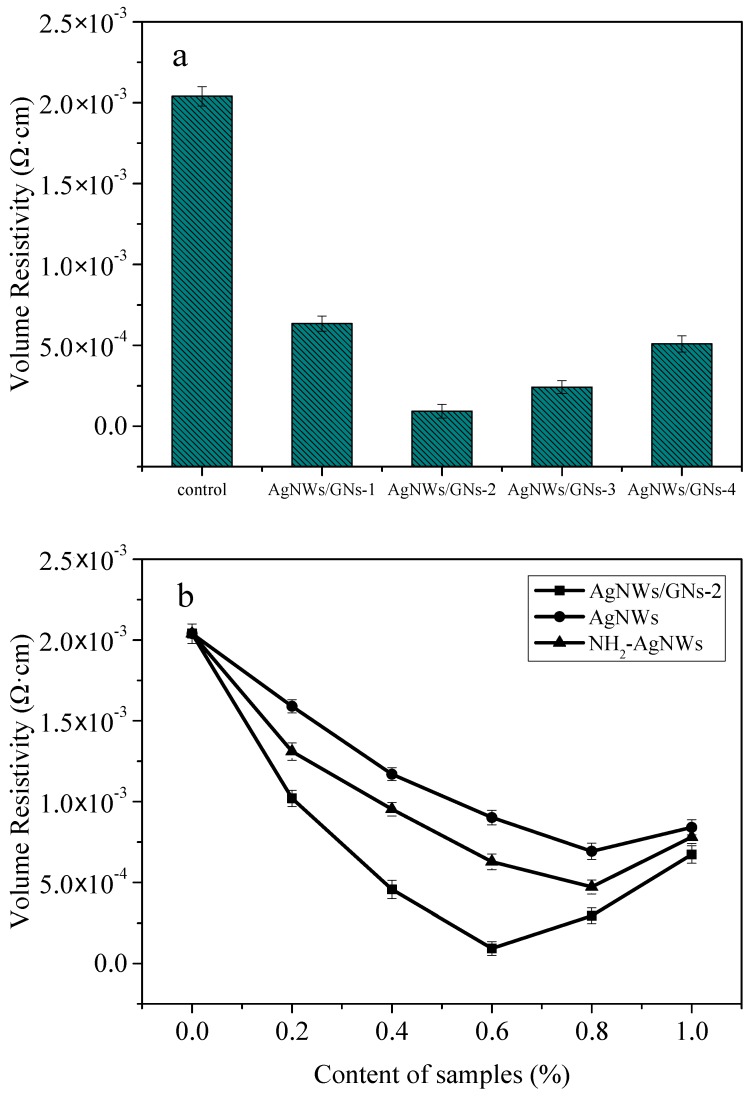
Volume resistivity of the ECAs filled with the nanocomposites with different proportions of AgNWs to GO (**a**) and different contents of the samples (**b**).

Figure 10 shows the impact of mass ratio of the silver nanowires to graphite oxide and the content of nanofiller on the conductive colloid product. As shown in Figure 10a, the volume resistivity of the conductive paste without adding any nanofiller was 2.04 × 10^−3^ Ω·cm. When the mass ratio increases, the volume resistivity of the conductive paste decreases first and then increases. When the mass ratio is 4:1 (AgNWs/GNs-2), the volume resistivity reaches a minimum value of 9.31 × 10^−5^ Ω·cm, which is 95.4% lower than the blank paste. This result can be explained by two reasons. First, AgNWs/GNs hybrids generate a continuous conductive pathway. Second, silver nanowires also serve as conductive paths between the sheets. However, when the content of silver nanowires lines is too high, agglomeration occurs in the silver nanowires lines, resulting in an increase in contact points and decrease in the conductivity. When the mass ratio of silver nanowires to graphite oxide is fixed at 4:1, adding AgNWs, NH_2_-AgNWs, and AgNWs/GNs-2 nanofillers can effectively improve the conductivity of the conductive paste. As shown in Figure 10b, when the content of the nanofillers increases, the volume resistivity of the conductive paste decreases first and then increases. When 0.6% AgNWs/GNs-2 hybrid is added, the volume resistivity reaches a minimum of 9.31 × 10^−5^ Ω·cm, which is reduced by about 95.4% compared with blank paste. When AgNWs and NH_2_-AgNWs with a mass fraction of 0.8% are added, the volume resistivity of the conductive paste can research 6.93 × 10^−4^ Ω·cm and 4.72 × 10^−4^ Ω·cm, respectively. During the curing process of the epoxy resin, the NH2 on the surface of the functionalized silver nanowires chemically reacted with the epoxy resin and formed chemical bonds, which can enhance the conductivity of the conductive paste.

## 4. Conclusions

This paper implemented self-assembly method to prepare the composite of silver nanowires and graphene as the filler of epoxy resin conductive adhesive. The structure and morphology of the composites were investigated and their impacts on the shear strength and electrical conductivity of the conductive adhesives were also studied. AgNWs play a crucial role not only in increasing electrical conductivity, but also in hindering GNs from restacking and aggregation. The reduced graphene (GNs) with surface functional groups can improve the mechanical performances of ECAs due to the chemical reaction with polymers and reduced percolation limit of fillers because of the uniform dispersion in the polymer matrix.

(1) Regarding the structure of the silver nanowires/graphene composite, the silver nanowires are uniformly dispersed on the surface of the graphene sheet layer. Such distribution can effectively form a two-dimensional nanocomposite structure and prevent graphene sheets from stacking.
(2) Adding silver nanowires/graphene composite can effectively improve the electrical conductivity and mechanical properties of the conductive adhesive. When the mass ratio of silver nanowires to graphite oxide is 4:1 and the mass fraction of the filled composite is 0.8%, the volume resistivity can reach the minimum of 9.31 × 10^−5^ Ω·cm, which is 95.4% lower than that of the reference sample. When the mass ratio of silver nanowires to graphite oxide is 6:1 and the mass fraction of the filled composite is 0.6%, the shear strength can reach the maximum of 14.3 MPa, which is 68.2% higher than that of the reference sample.

## Figures and Tables

**Table 1:** Summary of raw materials.

Materials	Supplier
Mercaptoethylamine (β-cysteamine)	Aladdin Chemical Co. Ltd. (Shanghai, China)
graphite oxide (GO)	Aladdin Chemical Co. Ltd. (Shanghai, China)
Hydrazine hydrate	Damao Chemisty Co. Ltd. (Tianjin, China)
Ethanediol	Damao Chemisty Co. Ltd. (Tianjin, China)
AgNO_3_	Sinopharm Chemical Reagent Co. Ltd. (Shanghai, China)
Polyvinylpyrrolidone (PVP)	Sinopharm Chemical Reagent Co. Ltd. (Shanghai, China)
FeCl_3_	Guangdong Guanghuang Chemical Reagent Co. Ltd. (Shantou, China)
Absolute ethyl alcohol	Guangdong Guanghuang Chemical Reagent Co. Ltd. (Shantou, China)
Acetone	Guangzhou Chemical Reagent Factory (Guangzhou, China)
MeH-HPA	Shanghai Macklin Bioc-tech Inc. (Shanghai, China)
2-Ethyl-4-methylimiadazole (2E4MZ)	Shanghai Macklin Bioc-tech Inc. (Shanghai, China)
Bisphenol-A epoxy resin (DGEBA, E-51, epoxy value = 0.54 mol/g)	Shanghai Balin petrochemical epoxy resin Co. Ltd. (Shanghai, China)

**Table 2:** Experimental equipment.

Equipment	Model	Manufacturer
Electric thermostat blast drying oven	DHG-9240A	Shanghai Shenxian Thermostatic Equipment (Shanghai, China)
Vacuum drying oven	DZF-6050	Shanghai Shenxian Thermostatic Equipment (Shanghai, China)
Ultrasonic cleaner	KII2200	Kunshan Hechuang Ultrasonic Instrument (Suzhou, China)
Electric blender	JJ-1A	Changzhou Aohua Instrument Co., Ltd. (Changzhou, China)
Constant temperature magnetic stirrer	Feb-85	Shanghai Sile Instrument Co., Ltd. (Shanghai, China)
High speed desktop centrifuge	TG1650-WS	Xiangyi Centrifuge Instrument (Changsha, China)
Scanning electron microscopy (SEM)	Merlin	Zeiss, Germany (Oberkochen, Germany)
Transmission electron microscopy (TEM)	JEM-2100F	Japan Electronics Corporation (Tokyo, Japan)
Automatic X-ray diffractometer	AXS D8	Bruker, Germany (Karlsruhe, Germany)
Fourier transform infrared spectrometer	Equinox-55	Bruker, Germany (Karlsruhe, Germany)
Raman spectrometer	LabRAM Aramis	H.J.Y, France (Paris, France)
X-ray photoelectron spectrometer	Axis Ultra	Shimadzu Kratos, Japan (Kyoto, Japan)
Electronic universal testing machine	AG-IC50kN	Suzhou Shimading Instrument Co., Ltd. (Suzhou, China)
Double electric logging four-point probe tester	RTS-9	Beijing Jinshisu Instrument Equipment Co., Ltd. (Beijing, China)

## References

[1] Tang T., Zhang X., Xu Z. (2010). Research progress and trends of electronic packaging materials. J. Nanjing Univ. Technol. (Nat. Sci. Ed.).

[2] Huang Q., Gu M., Jin Y. (2000). Current Status of Research on Electronic Packaging Materials. Mater. Rev..

[3] Li Y., Wong C.P. (2006). Recent advances of conductive adhesives as a lead-free alternative in electronic packaging: Materials, processing, reliability and applications. Mater. Sci. Eng. R Rep..

[4] Yim M.J., Li Y., Moon K., Paik K.W., Wong C.P. (2008). Review of recent advances in electrically conductive adhesive materials and technologies in electronic packaging. J. Adhes. Sci. Technol..

[5] He Y. (2008). The Study and Development of Electrically Conductive Adhesive. Chem. Adhes..

[6] Li Y., Yim M.J., Moon K.S., Wong C.P. (2009). Novel nano-scale conductive films with enhanced electrical performance and reliability for high performance fine pitch interconnect. IEEE Trans. Adv. Packag..

[7] Li Y., Moon K.S., Wong C.P. (2005). Electronics without lead. Science.

[8] Ramkumar S.M., Srihari K. (2007). A novel anisotropic conductive adhesive for lead-free surface mount electronics packaging. J. Electron. Packag..

[9] Park B.K., Jeong S., Kim D., Moon J., Lim S., Kim J.S. (2007). Synthesis and size control of monodisperse copper nanoparticles by polyol method. J. Colloid Interface Sci..

[10] Li H.Y., Moon K.S., Wong C.P. (2004). A novel approach to stabilize contact resistance of electrically conductive adhesives on lead-free alloy surfaces. J. Electron. Mater..

[11] Li Y., Moon K.S., Wong C.P. (2005). Monolayer-protected silver nano-particle-based anisotropic conductive adhesives: Enhancement of electrical and thermal properties. J. Electron. Mater..

[12] Tolvgard A., Malmodin J., Liu J., Lai Z. (1999). A reliable and environmentally friendly packaging technology-flip-chip joining using anisotropically conductive adhesive. IEEE Trans. Compon. Packag. Technol..

[13] Jiang H., Moon K., Li Y., Wong C. (2006). Surface functionalized silver nanoparticles for ultrahigh conductive polymer composites. Chem. Mater..

[14] Wu H.P., Wu X.J., Ge M.Y., Zhang G.Q., Wang Y.W., Jiang J.Z. (2007). Effect analysis of filler sizes on percolation threshold of isotropical conductive adhesives. Compos. Sci. Technol..

[15] Zhang R., Moon K.S., Lin W., Wong C.P. (2010). Preparation of highly conductive polymer nanocomposites by low temperature sintering of silver nanoparticles. J. Mater. Chem..

[16] Lee H.H., Chou K.S., Shih Z.W. (2005). Effect of nano-sized silver particles on the resistivity of polymeric conductive adhesives. Int. J. Adhes. Adhes..

[17] Gao H., Liu L., Luo Y., Jia D.M. (2011). In-situ preparation of epoxy/silver nanocomposites by thermal decomposition of silver-imidazole complex. Mater. Lett..

[18] Yang C., Wong C.P., Yuen M.M.F. (2013). Printed electrically conductive composites: Conductive filler designs and surface engineering. J. Mater. Chem. C.

[19] Polavarapu L., Manga K.K., Cao H.D., Loh K.P., Xu Q.H. (2011). Preparation of conductive silver films at mild temperatures for printable organic electronics. Chem. Mater..

[20] Akter T., Kim W.S. (2012). Reversibly stretchable transparent conductive coatings of spray-deposited silver nanowires. ACS Appl. Mater. Interfaces.

[21] Wu H.P., Liu J.F., Wu X.J., Ge M.Y. (2006). High conductivity of isotropic conductive adhesives filled with silver nanowires. Int. J. Adhes. Adhes..

[22] Yu Y., Ma C.M., Yuen S., Teng C., Huang Y., Wang I., Wei M. (2010). Morphology, electrical, and rheological properties of silane-modified silver nanowire/polymer composites. Macromol. Mater. Eng..

[23] Chen C., Wang L., Li R., Jiang G., Yu H., Chen T. (2007). Effect of silver nanowires on electrical conductance of system composed of silver particles. J. Mater. Sci..

[24] Chen D., Qiao X., Qiu X., Tan F., Chen J., Jiang R. (2010). Effect of silver nanostructures on the resistivity of electrically conductive adhesives composed of silver flakes. J. Mater. Sci. Mater. Electron..

[25] Wang Y.H., Xiong N.N., Li Z.L., Xie H., Liu J.Z., Dong J., Li J.Z. (2015). A comprehensive study of silver nanowires filled electrically conductive adhesives. J. Mater. Sci. Mater. Electron..

[26] Ma H., Zeng J., Harrington S., Ma L., Ma M., Guo X., Ma Y. (2016). Hydrothermal fabrication of silver nanowires-silver nanoparticles-graphene nanosheets composites in enhancing electrical conductive performance of electrically conductive adhesives. Nanomaterials.

[27] Zhang Z.X., Chen X.Y., Xiao F. (2011). The sintering behavior of electrically conductive adhesives filled with surface modified silver nanowires. J. Adhes. Sci. Technol..

[28] Novoselov K.S., Geim A.K., Morozov S.V., Jiang D., Katsnelson M.I., Grigorieva I.V., Grigorieva S.V., Dubonos S.V., Firson A.A. (2005). Two-dimensional gas of massless Dirac fermions in graphene. Nature.

[29] Lu X., Jin Y., Tan S., Zhang L., Liu Y., Zhang X., Xu J. (2008). A simple approach for fabricating a superhydrophobic surface based on poly(methyl methacrylate). J. Adhes. Sci. Technol..

[30] Wajid A.S., Ahmed H.S.T., Das S., Irin F., Jankowski A.F., Green M.J. (2013). High-performance pristine graphene/epoxy composites with enhanced mechanical and electrical properties. Macromol. Mater. Eng..

[31] Marcq F., Demont P., Monfraix P., Peigney A., Laurent Ch., Falat T., Courtade F., Jamin T. (2011). Carbon nanotubes and silver flakes filled epoxy resin for new hybrid conductive adhesives. Microelectron. Reliab..

[32] Luan V.H., Tien H.N., Cuong T.V., Kong B.S., Chung J.S., Kim E.J., Hur S.H. (2012). Novel conductive epoxy composites composed of 2-D chemically reduced graphene and 1-D silver nanowire hybrid fillers. J. Mater. Chem..

[33] Oh Y., Chun K.Y., Lee E., Kim Y.J., Baik S. (2010). Functionalized nano-silver particles assembled on one-dimensional nanotube scaffolds for ultra-highly conductive silver/polymer composites. J. Mater. Chem..

[34] Wu H.P., Wu X.J., Ge M.Y., Zhang G.Q., Wang Y.W., Jiang J. (2007). Properties investigation on isotropical conductive adhesives filled with silver coated carbon nanotubes. Compos. Sci. Technol..

[35] Amoli B.M., Trinidad J., Hu A., Zhou Y.N., Zhao B. (2015). Highly electrically conductive adhesives using silver nanoparticle (Ag NP)-decorated graphene: The effect of NPs sintering on the electrical conductivity improvement. J. Mater. Sci. Mater. Electron..

[36] Chen D., Qiao X., Qiu X., Chen J., Jiang R. (2010). Convenient synthesis of silver nanowires with adjustable diameters via a solvothermal method. J. Colloid Interface Sci..

[37] Tien H.W., Hsiao S.T., Liao W.H., Yu Y.H., Lin F.C., Wang Y.S., Li S.M., Ma C.C.M. (2013). Using self-assembly to prepare a graphene-silver nanowire hybrid film that is transparent and electrically conductive. Carbon.

[38] Techane S.D., Gamble L.J., Castner D.G. (2011). X-ray photoelectron spectroscopy characterization of gold nanoparticles functionalized with amine-terminated alkanethiols. Biointerphases.

[39] Sookhakian M., Ridwan N.A., Zalnezhad E., Yoon G.H., Azarang M., Mahmoudian M.R., Alias Y. (2016). Layer-by-layer electrodeposited reduced graphene oxide-copper nanopolyhedra films as efficient platinum-free counter electrodes in high efficiency dye-sensitized solar cells. J. Electrochem. Soc..

[40] Pimenta M.A., Dresselhaus G., Dresselhaus M.S., Cancado L.G., Jorio A., Saito R. (2007). Studying disorder in graphite-based systems by Raman spectroscopy. Phys. Chem. Chem. Phys..

[41] Stankovich S., Dikin D.A., Piner R.D., Kohlhaas K.A., Kleinhammes A., Jia Y., Wu Y., Nguyen S.T., Ruoff R.S. (2007). Synthesis of graphene-based nanosheets via chemical reduction of exfoliated graphite oxide. Carbon.

[42] Prolongo S.G., Campo M., Gude M.R., Chaos-Morán R., Ureña A. (2009). Thermo-physical characterisation of epoxy resin reinforced by amino-functionalized carbon nanofibers. Compos. Sci. Technol..

[43] Wang J., Fang Z., Gu A., Xu L., Liu F. (2006). Effect of amino-functionalization of multi-walled carbon nanotubes on the dispersion with epoxy resin matrix. J. Appl. Polym. Sci..

